# Prevalence of extended spectrum β-lactamase and carbapenemase-producing *Escherichia coli* and *Klebsiella pneumoniae* in raw bulk cow milk from dairy cooperatives, Northwest Amhara, Ethiopia

**DOI:** 10.1371/journal.pone.0336987

**Published:** 2025-11-14

**Authors:** Chalachew Genet, Wendemagegn Enbiale, Anna Rommerskirchen, Rajiha Abubeker, Wudu Tafere, Tsehaynesh Gebre-Eyesus, Michael Getie, Alem Tsega, Muluken Acham, Addisu Melese, Tewachew Awoke, Wondemagegn Mulu, Degu Ashagrie, Tadele Amsalu, Achenef Motbainor, Endalew Gebeyehu, Mulugeta Kibret, Bayeh Abera, Endalkachew Nibret, Abaineh Munshea

**Affiliations:** 1 Health Biotechnology Division, Institute of Biotechnology, Bahir Dar University, Bahir Dar, Ethiopia; 2 Department of Medical Microbiology, Bahir Dar University, Bahir Dar, Ethiopia; 3 Department of Dermatovenereology, Bahir Dar University, Bahir Dar, Ethiopia; 4 Training Center for Neglected Tropical Disease, Arba Minch University, Arba Minch, Ethiopia; 5 Institute of Medical Microbiology and Hospital Hygiene, Heinrich-Heine-University, Düsseldorf, Germany; 6 Hirsch Institute of Tropical Medicine, Asella University, Asella, Ethiopia; 7 Ethiopian Public Health Institute, Addis Ababa, Ethiopia; 8 Amhara Public Health Institute, Bahir Dar, Ethiopia; 9 Laboratory of Microbiology, Department of Biochemistry and Microbiology, Ghent University, Ghent, Belgium; 10 Diagnostic Medical Microbiology Unit, Felege-Hiwot Comprehensive Specialized Hospital, Bahir Dar, Ethiopia; 11 Diagnostic Medical Microbiology Unit, Tibebe-Ghion Specialized Hospital, Bahir Dar, Ethiopia; 12 School of Public Health, Bahir Dar University, Bahir Dar, Ethiopia; 13 Department of Pharmacy, Bahir Dar University, Bahir Dar, Ethiopia; 14 Department of Biology, Bahir Dar University, Bahir Dar, Ethiopia; Hawassa University College of Medicine and Health Sciences, ETHIOPIA

## Abstract

**Introduction:**

Extended spectrum β-lactamase (ESBL) and carbapenemase-producing *Escherichia coli* (*E. coli*) and *Klebsiella pneumoniae* (*K. pneumoniae*) emanating from raw cow milk are among the leading contributors to the spread of antimicrobial resistance (AMR). Due to the misuse and overuse of antibiotics in dairy farms, cow’s milk has become a reservoir of ESBL- and carbapenemase-producing *E. coli* and *K. pneumoniae* posing a growing public health threat, especially in areas where the consumption of raw milk is common. However, compared to the clinical sector, the prevalence of ESBL- and carbapenemase-producing *E. coli* and *K. pneumoniae* in the food sector is under-studied.

**Objective:**

This study aimed to determine the prevalence of ESBL and carbapenemase-producing *E. coli* and *K. pneumoniae* in raw bulk cow milk from Dairy Cooperatives in Northwest Amhara, Ethiopia.

**Methods:**

A cross-sectional study was conducted from January to April, 2025 among 257 dairy cooperative member farms. Sociodemographic and related data were collected using a structured questionnaire. Five milliliters of raw bulk cow milk were collected aseptically from each farm in four Dairy Cooperatives (DCs) (DC-A to D). 10 microliters of milk sample were directly inoculated into MacConkey agar. *Escherichia coli* and *K. pneumoniae* were identified using standard microbiological techniques. Antimicrobial susceptibility testing was performed using the Kirby-Bauer disk diffusion method. ESBL and carbapenemase production were confirmed phenotypically via combination disk tests and modified carbapenem inactivation methods, respectively.

**Results:**

The prevalence of *E. coli* and/or *K. pneumoniae* in raw cow milk was 21% (95% CI, 16.5–26.4%), with respective individual prevalence of 8.2% and 14.8%. ESBL-producing *E. coli* and *K. pneumoniae* accounted for 23.8% and 15.8% of isolates, respectively, while 2.6% of isolates (only *K. pneumoniae*) were carbapenemase producers. Resistance to ampicillin and amoxicillin-clavulanic acid exceeded 70%. All *E. coli* and 94.7% of *K. pneumoniae* isolates remained susceptible to carbapenems. Nearly half of all isolates (45.8%) were multidrug resistant (MDR), and 51.9% of MDR isolates were co-resistant to at least six antibiotics. Having additional non-farming occupations (AOR: 4.17, 95% CI: 1.49–11.67), large herd size (AOR: 3.21, 95% CI: 1.26–8.18), having pet animals (AOR: 6.53, 95% CI: 1.39–30.7), and use of calabash milk pail (AOR: 7.37, 95% CI: 1.45–37.49) were significantly associated with milk culture positive result for *E. coli* and/or *K. pneumoniae.*

**Conclusion:**

Raw milk in Northwest Amhara harbors ESBL and carbapenemase-producing *E. coli* and *K. pneumoniae* posing a substantial public health risk coupled with MDR and resistance to critically important antimicrobials. Strengthened AMR surveillance, improved farm hygiene, restricted antibiotic use, and public education on milk safety are urgently needed.

## Introduction

Antimicrobial resistance (AMR) has emerged as one of the most pressing global health challenges of the 21^st^ century, with profound implications for both human and veterinary medicine globally [1]. Antimicrobial resistance compromises the effectiveness of treatments for bacterial infections, leading to prolonged illness, increased healthcare costs, and higher mortality rates. In 2021 alone, AMR was linked to an estimated 4.71 million human deaths, with projections, this figure may rise to 8.22 million by 2050 in the absence of a robust intervention strategy [2]. Among the bacterial pathogens contributing most significantly to this burden are extended-spectrum cephalosporins and carbapenem-resistant *Escherichia coli* (*E. coli*) and *Klebsiella pneumoniae* (*K. pneumoniae*) [2].

The prevalence of extended spectrum cephalosporin and carbapenem-resistant *E. coli* and *K. pneumoniae* due to extended spectrum β-lactamase (ESBL) and carbapenemase production, respectively [3,4] is increasing among human, animal and environmental isolates complicating their public health impact [5,6]. ESBL and carbapenemase-producing strains of *E. coli* and *K. pneumoniae* are a growing significant cause of invasive infections such as septicemia and urinary tract infection in humans [7] as well as mastitis in cows [8,9]. Such infection due to resistant strains result in a higher mortality rate than infections due to their susceptible counterparts [7,10]. An increase in ESBL and carbapenemase-producing *E. coli* and *K. pneumoniae* is partly attributed to misuse and an ever-increasing use of antibiotics in health facilities [11], community [12] and dairy farms [13,14] for treatment, and prophylaxis [15], especially in developing countries [14,16,17].

Though there are international and national recommendations and guidelines on the prevention and control of AMR in animals and animal foods [18–20] data on ESBL and carbapenemase-producing *E. coli* and *K. pneumoniae* from animal food sources including raw cow milk is scarce [21].

Cow milk, while highly nutritious and beneficial to human health [22,23] also presents a risk when consumed row [24–27]. Cow milk is mainly consumed in pasteurized form [28] but consumption of raw cow milk is a common practice in many low-income settings including Ethiopia [29,30]. This is due to lack of facilities for pasteurization coupled with widespread tradition that heating cow milk will cause loss of its nutritional value and taste [31].

Ethiopia, home to the largest livestock population in Africa [32], faces multiple challenges in addressing AMR, including inadequate antibiotic stewardship, lack of awareness among livestock handlers, and weak regulatory frameworks [33].

Despite these challenges, and the high consumption of raw cow milk and dairy products in Ethiopia, limited data exist on the prevalence and resistance profiles of ESBL and carbapenemase-producing *E. coli* and *K. pneumoniae* in milk. This data gap hampers the ability of stakeholders including dairy cooperatives, public health authorities, and researchers to implement evidence-based interventions [19,34]. Therefore, this study was undertaken to determine the prevalence of ESBL- and carbapenemase-producing *E. coli* and *K. pneumoniae* in raw bulk cow milk collected from dairy cooperatives, in the northwest part of Amhara Regional State, Ethiopia.

## Materials and methods

### Ethics statement

The study was ethically approved by Bahir Dar University Institute of Biotechnology Institutional Research Ethics Review Committee with reference number IoB-IRERC/001/2024. Verbal informed consent was secured from all participants before milk sampling and sociodemographic data collection. Data confidentiality and anonymity were strictly maintained throughout the study.

### Study design, area, and period

A cross sectional study was conducted between January to April, 2025 among dairy development and marketing cooperative member farms located in Bahir Dar Special Zone and North Gojjam Zone located in the Northwest part of Amhara Regional State, Ethiopia. In Bahir Dar Special Zone, there are four Dairy development and marketing cooperatives named Bahir Dar, Tis-Abay, Tiret and Zenzelma dairy development and marketing cooperatives. Meanwhile, there are seven dairy development and marketing cooperatives in North Gojjam Zone Bahir Dar Zuria Woreda named Abadama, Abay Zuria, Addis-Alem, Emfraz, Felege-Ghion, Genet Lerobit, and Tseyit dairy cooperatives. These dairy cooperatives supply unprocessed raw cow milk to residents of Bahir Dar city and North Gojjam Zone through cooperative outlets, private milk vendors, and house-to-house delivery systems. In addition to raw milk, they process and distribute milk-derived products such as butter, cheese, and yogurt, with some shipments sent to Addis Ababa, Ethiopia.

Dairy development and marketing cooperatives such as Bahir Dar, Tiret and Zenzelma have or are working to have milk processing plants in Bahir Dar city, part of our study area. Bahir Dar city is the capital city of Amhara Regional State located at 11°37′ N and 37°25′ E 565 Kilometers away from the country’s capital Addis Ababa via Bure district [35]. The city showed a temperature rise at a higher rate than the global average. Meanwhile, the city had relatively higher humidity than the nearby city in the region [36].

### Sample size and sampling techniques

A total of 257 consecutively selected dairy development and marketing cooperative member farms currently providing raw cow milk to the cooperatives were included using a single population proportion formula assuming 21.2% prevalence of ESBL-producing *E. coli* in bulk tank milk [25], a 5% margin of error and 95% level of confidence. The total sample size was proportionally distributed to four actively operating dairy development and marketing cooperatives (referred to Dairy cooperative-A (DC-A), Dairy cooperative-B (DC-B), Dairy cooperative-C (DC-C) and Dairy cooperative-D (DC-D) for confidentiality) in the study area based on the number of their active members during the study period (Table 1).

**Table 1:** Proportional distribution of the total sample size to four dairy development and marketing cooperatives included in this study, in Northwest Ethiopia, 2025.

DC name	Number of DC active members	Number of DC member farms selected
DC-A	180	104
DC-B	124	72
DC-C	95	55
DC-D	45	26
Total	444	257

Note: DC→ Dairy Cooperatives

### Operational definition

**Dairy cooperative member**: An individual who owns a dairy farm and is registered as a member in one of the four dairy development and marketing cooperatives included in this study and provides milk to the cooperative during the study period.

**Dairy farm size**: categorized as “large farm” if it has more than 10 cattle and “small farm” if it has 10 or fewer cattle [37].

**Northwest Amhara**: In this study, it refers Bahir Dar Special Zone and the North Gojjam Zone.

**Pet animal**: In this study, pet animal refers to dogs and/or cats.

**Prevalence of ESBL-producing *E. coli* and/or *K. pneumoniae***: this was determined by dividing ESBL-producing *E. coli* and/or *K. pneumoniae* by their respective total isolates screened.

**Prevalence of carbapenemase-producing *E. coli* and/or *K. pneumoniae***: This was determined by dividing carbapenemase-producing *E. coli* and/or *K. pneumoniae* by their respective total isolates screened.

### Sociodemographic and related data collection

Data on sociodemographic variables such as age, sex, family size and educational level of dairy farm owners along with farm-related practices including herd size, free grazing, presence of pet animals, and antibiotic use practice for animals were collected using a structured questionnaire administered by trained data collectors.

### Raw cow bulk milk sample collection

Following previously established protocol [38], raw bulk cow milk samples were collected from four dairy development and marketing cooperatives by visiting their milk collection centers. Briefly, 5 ml of raw bulk cow milk from milk buckets of selected DC members was collected aseptically using a sterile syringe before bulking to the DC milk tank. The collected milk was transferred immediately to a single-use 50 ml Falcon tube and delivered within three hours to the Environmental Health and Biomedical Reference Laboratory of Amhara Public Health Institute, Bahir Dar, Ethiopia in a cold box for processing.

### Bacterial isolation and identification

*Escherichia coli* and *K. pneumoniae* were identified following standard bacteriological techniques as described in WHO guideline [39] and a prior study [40]. A 10µl aliquot of raw bulk milk samples was directly inoculated into MacConkey agar plate (HiMedia, India) and incubated at 37^o^C aerobically for 24 hours. Colony with morphology suggestive of *E. coli* (smooth circular pink colony) and *K. pneumoniae* (mucoid pink colony) were subcultured into blood agar plate and incubated at 37^o^C for 24 hours. Then, colony on blood agar was further tested using a panel of biochemical assays to assess characteristics such as carbohydrate fermentation, motility, hydrogen sulfide production, citrate permease, lysine decarboxylase and urease production.

### Antimicrobial susceptibility testing

Antimicrobial susceptibility testing was performed using the modified Kirby-Bauer disc diffusion method following the Clinical Laboratory Standard Institute (CLSI) guideline [41]. A total of 14 antibiotics selected based on prior studies [25,42,43], and their use in Ethiopia [40,44] including ampicillin (AMP, 10 µg), cefazolin (KZ, 30 µg), cefuroxime (CXM, 30 µg), ceftazidime (CAZ, 30 µg), cefotaxime (CTX, 30 µg), meropenem (MRP, 10 µg), imipenem (IPM, 10 µg), gentamicin (GM, 10 µg), ciprofloxacin (CIP, 5 µg), chloramphenicol (C, 30 µg), tetracycline (TE, 30 µg), amoxycillin-clavulanic acid (AMC, 30 µg), amikacin (AK, 30 µg), Co-Trimoxazole (COT, 25 µg). Except for the last three, which were obtained from HiMedia, India, all antibiotics were from Oxoid (Oxoid, Hampshire, United Kingdom). *E. coli* and *K. pneumoniae* resistant to at least three antibiotics in different antibiotic groups were considered multidrug resistant (MDR) [45].

### Detection of ESBL-producing *E. coli* and *K. pneumoniae*

Phenotypic screening and confirmation of ESBL production were done for all *E. coli* and *K. pneumoniae* isolates based on the CLSI M100 guideline [41]. Isolates showing an inhibition zone of ≤ 22 mm, and/or ≤ 27 mm for ceftazidime, and cefotaxime, respectively were considered as suspect for ESBL production and further confirmed phenotypically by the combination disc diffusion test. Briefly, isolates suspected as ESBL producers were tested against ceftazidime (30 μg), ceftazidime-clavulanic acid (30/10 μg), cefotaxime (30 μg) and cefotaxime-clavulanic acid (30/10 μg) discs using the Kirby-Bauer disc diffusion method on Muller-Hinton Agar (MHA, HiMedia, India). After incubating at 37^o^C for 18 hours, an increase of 5 or more mm in zone of inhibition for either of the antimicrobial agents tested in combination with clavulanic acid than the zone diameter of the agents when tested alone was considered an ESBL producer.

Furthermore, ESBL-producing *E. coli* and *K. pneumoniae* isolates were tested against six antibiotics considered as a possible carbapenem-sparing treatment option for ESBL-producing *E. coli* and *K. pneumoniae* [46,47] using disc diffusion methods [41]. The antibiotics tested include aztreonam (ATM, 30) cefepime (FEP, 30 μg), piperacillin-tazobactam (TZP, 110 μg), cefoxitin (FOX, 30 µg), cefotetan (CTT, 30 µg) and nitrofurantoin (NIT, 300 μg) obtained from Oxoid, UK except Nitrofurantoin from Micromaster, India.

### Detection of carbapenemase-producing *E. coli* and *K. pneumoniae*

Carbapenemase production was detected using the modified carbapenem inactivation method (mCIM) having more than 99% detection sensitivity and specificity following the CLSI M100 guideline [41]. Initial screening of all *E. coli* and *K. pneumoniae* isolates for carbapenemase production was done using the Kirby-Bauer disk diffusion method with meropenem (10 µg) and imipenem (10 µg) on MHA. If the inhibition zone for meropenem and/or imipenem is ≤ 19 mm, it was considered a suspect for carbapenemase production and further confirmed using mCIM. In this method, 1µl loopful colony of the suspected isolate from overnight incubated blood agar (HiMedia, Mumbai, India) was transferred into 2 ml Tryptone Soya Broth (TSB, HiMedia, India) followed by vortexing for 10 seconds. Then it was incubated for 4 hours at 37ºC after adding a meropenem (10 µg) disc. A 0.5 McFarland suspension of meropenem susceptible *E. coli* American Type Culture Collection (ATCC) 25922 was evenly swabbed on MHA and then the meropenem in the TSB was dispensed followed by incubation for 24 hours at 37ºC. The inhibition zone diameter of 6–15 mm or the presence of pinpoint colonies within a 16–18 mm inhibition zone indicated carbapenemase producer. Furthermore, *E. coli* and *K. pneumoniae* isolates identified as carbapenemase producer were further tested against two β-lactam/β-lactamase inhibitor combination antibiotics (ceftazidime-avibactam and meropenem-vaborbactam).

### Quality assurance

To maintain the quality of data collected, different quality assurance measures were implemented. Training was given to data collectors before data collection. The questionnaire was checked for completeness during and after data collection. The sterility test was performed by incubating 5% of un-inoculated bacterial culture media from each batch [39]. The *K. pneumoniae* American Type Culture Collection (ATCC) 700603 and *E. coli* ATCC 25922 were used as positive and negative controls for ESBL detection, respectively. Besides, *E. coli* ATCC 25922 and *K. pneumoniae* ATCC BAA1705 were used as negative and positive controls for carbapenemase production, respectively [41]. All reference strains were obtained from the Ethiopian Public Health Institute.

### Data analysis

All data were coded, entered and analyzed using SPSS version 25. Descriptive statistics were used to summarize key variables. Chi-square and Fisher’s exact test were used to see the presence of any association between variables. Bivariate analysis was performed, and those variables with a *p*-value < 0.25 were considered for multivariate analysis. *P*-values < 0.05 were considered significant [48].

## Results

### Socio-demographic and related characteristics of dairy cooperative members

Among 257 DC member farms included in the study, 218 (84.8%) and 230 (89.5%) were owned by male and married individuals, respectively. The age of dairy farm owners included in the study ranged from 20 to 95 years with an average age of 47.9 ± 11.7 years. More than half (52.5%) of the DC farm owners were aged 49 years or younger. None of the DC-D members attended school beyond the primary level, and all were rural residents. Two hundred forty-four (94.9%) and nearly half (110/257, 42.8%) of DC farm members had separate cow barns and employed at least 1 worker in their dairy farm, respectively. Furthermore, animal farming was the only source of income for 211 (82.1%) members (Table 2).

**Table 2:** Sociodemographic and farm facility and management-related characteristics of dairy development and marketing cooperative members in Northwest Ethiopia, 2025.

Variable	Name of dairy cooperatives: n^☼^ (%)			
	DC-A (n = 104)	DC-B (n = 72)	DC-C (n = 55)	DC-D (n = 26)
**Sex of DC members**				
Male	88 (84.6)	66 (91.7)	40 (72.7)	24 (92.3)
Female	16 (15.4)	6 (8.3)	15 (27.3)	2 (7.7)
**Age of DC members (in years)**				
15-49	63 (60.6)	37 (51.4)	18 (32.7)	17 (65.4)
> 49	41 (39.4)	35 (48.6)	37 (67.3)	9 (34.6)
**Marital status of DC members**				
Single	6 (5.8)	2 (2.8)	2 (3.6)	0 (0)
Married	93 (89.4)	68 (94.4)	47 (85.5)	22 (84.6)
Divorced	3 (2.9)	0 (0)	0 (0)	2 (7.7)
Widowed	2 (1.9)	2 (2.8)	6 (10.9)	2 (7.7)
**Educational status of DC members**				
Unable to read and write	42 (40.4)	40 (55.5)	9 (16.4)	14 (53.8)
Able to read and write	14 (13.5)	6 (8.3)	9 (16.4)	4 (15.4)
Primary school (grade: 1–8)	18 (17.3)	13 (18.1)	20 (36.3)	8 (30.8)
Above primary school	30 (28.8)	13 (18.1)	17 (30.9)	0 (0)
**Residence of DC members**				
Urban	35 (33.7)	6 (8.3)	52 (94.5)	0 (0)
Rural	69 (66.3)	66 (91.7)	3 (5.5)	26 (100)
**Occupation of DC members**				
Farming only	87 (83.7)	65 (90.3)	39 (70.9)	20 (76.9)
Have additional work^⁑^	17 (16.3)	7 (9.7)	16 (29.1)	6 (23.1)
**Family size of DC members**				
Small (≤ 5)	62 (59.6)	47 (65.3)	39 (70.9)	17 (65.4)
Large (> 5)	42 (40.4)	25 (34.7)	16 (29.1)	9 (34.6)
**Herd size of animals for DC members**				
Small (≤10 cattle)	91 (87.5)	59 (81.9)	27 (49.1)	22 (84.6)
Large	13 (12.5)	13 (18.1)	28 (50.9)	4 (15.4)
Number of lactating cows				
≤ 5 cows	103 (99)	70 (97.2)	45 (81.8)	26 (100)
> 5 cows	1 (1)	2 (2.8)	10 (18.2)	0 (0)
**Presence of cowherd in each dairy farm**				
Have at least 1 cowherd	41 (39.4)	30 (41.7)	32 (58.2)	7 (26.9)
No cowherd	63 (60.6)	42 (58.3)	23 (41.8)	19 (73.1)
**Domestic animal type**				
Cattle only	42 (40.4)	23 (31.9)	27 (49.1)	8 (30.8)
Cattle and other animals^♦^	62 (59.6)	49 (68.1)	28 (50.9)	18 (69.2)
**Have separate cow barn**				
Yes	98 (94.2)	71 (98.6)	53 (96.4)	22 (84.6)
No	6 (5.8)	1 (1.4)	2 (3.6)	4 (15.4)
**Buy animal drugs without prescription**				
Yes	104 (100)	72 (100)	55 (100)	26 (100)
No	0	0	0	0

**Note**: ⁑ → DC-A→ Dairy Cooperative-A, DC-B→ Dairy Cooperative-B, DC-C→ Dairy Cooperative, DC-D→ Dairy Cooperative-D. It includes involvement in one of the other works namely merchant, government employee, or employee in private organization. ♦ → other animals include 1 or more sheep, goat, donkey, mule, and hen, ☼ → “n” indicates the number of dairy farm owners which provide their raw bulk cow milk.

The average milk supplied to the DCs by each member farm included in this study per day/lactating cow was 6.9 ± 3.1 liters. The highest and the lowest amount of milk/cow/day was collected by DC-C (9.1 liters ± 3.1) and DC-D (5.7 liters ± 2.3), respectively. Out of 5, 058 liters collected each day by four DCs from their members included in this study, the highest amount was collected by DC-C (2, 296 liters) followed by DC-A (1, 274 liters), DC-B (1, 193 liters) and DC-D (295 liters).

### Prevalence of *E. coli* and *K. pneumoniae* in raw bulk cow milk

**Fig 1:**
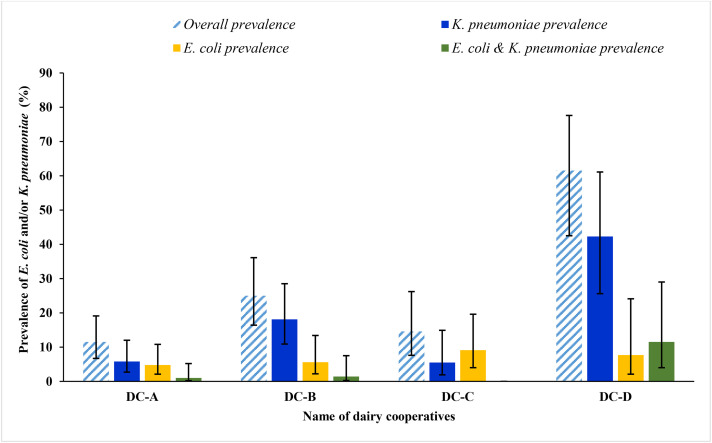
Prevalence of *E. coli* and/or *K. pneumoniae* among raw bulk cow milk from dairy development and marketing cooperative member farms in Northwest Ethiopia, 2025. **Note**: The error bars in each graph indicate the 95% confidence interval for point prevalence. DC-A→ Dairy Cooperative-A, DC-B→ Dairy Cooperative-B, DC-C→ Dairy Cooperative, DC-D→ Dairy Cooperative-D.

The prevalence of *E. coli* and/or *K. pneumoniae* in bulk cow milk was 54/257, 21% (95% CI, 16.5–26.4%) with 21/257, 8.2% (95% CI, 5.4–12.2%) and 38/257, 14.8% (95% CI, 11–19.7%) prevalence for *E. coli* and *K. pneumoniae,* respectively. Both *E. coli* and *K. pneumoniae* were concurrently isolated from 5 of culture-positive raw bulk cow milk samples (5/54, 9.3%) across the three DCs. The prevalence of *E. coli* and/or *K. pneumoniae* showed significant variation among DCs, ranging from 11.5% to 61.5% (chi-square *p*-value < 0.001). Of the total 59 bacteria isolated, 38 (64.4%) were *K. pneumoniae*, the dominant isolate in three DCs. The majority of *K. pneumoniae* (73.7%) were isolated from DC-B and DC-D. Except for DC-C, *E. coli* and *K. pneumoniae* were identified concurrently in all dairy cooperatives (Fig 1).

### Prevalence of ESBL and carbapenemase-producing *E. coli* and *K. pneumoniae*

**Fig 2:**
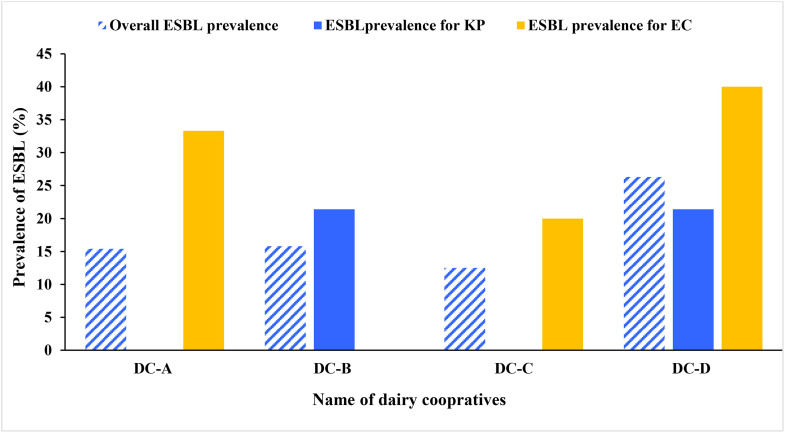
Prevalence of ESBL-producing *E. coli* and *K. pneumoniae* isolated from raw bulk cow milk from dairy development and marketing cooperative member farms in Northwest Ethiopia, 2025. **Note**: ESBL→ Extended Spectrum β-lactamase, EC → *E. coli*, KP → *K. pneumoniae.* DC-A→ Dairy Cooperative-A, DC-B→ Dairy Cooperative-B, DC-C→ Dairy Cooperative, DC-D→ Dairy Cooperative-D.

The overall ESBL-producing *E. coli* and/or *K. pneumoniae* prevalence was 18.6% (11/59, 95% CI: 10.7–30.4). The prevalence showed significant variation among dairy cooperatives (Fisher’s exact test *p* < 0.001), with the highest and the lowest being recorded from DC-D (5/19, 26.3%) and DC-C (1/8, 12.5%), respectively. The prevalence of ESBL-producing *E. coli* and *K. pneumoniae* alone was 23.8% (5/21, 95% CI: 10.6–45.1%) and 15.8% (6/38, 95% CI: 7.4–30.4%), respectively. Half of ESBL-producing *K. pneumoniae,* with no ESBL-producing *E. coli,* were detected in DC-B (Fig 2).

The overall prevalence of carbapenemase-producing *E. coli* and/or *K. pneumoniae* in raw bulk cow milk was 2.6% (1/38, 95% CI: 0.5–13.5). Among 59 bacteria isolates, only 1 carbapenemase-producing *K. pneumoniae* was isolated from a milk sample collected from a widowed male dairy farm owner who is a member of DC-B and has a large herd size. This isolate was resistant to all 21 antibiotics tested (except chloramphenicol), including ceftazidime-avibactam and meropenem-vaborbactam combination antibiotics.

**Fig 3:**
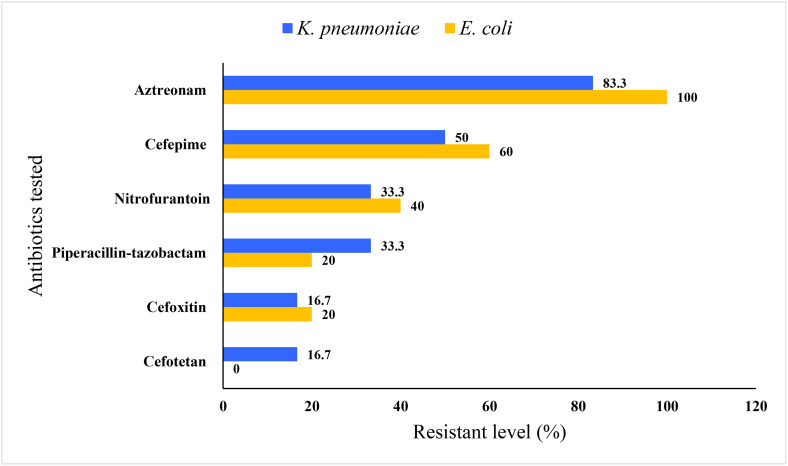
Antibiotic resistance profile of ESBL producing *E. coli* and *K. pneumoniae* against selected carbapenem-sparing antibiotics, Northwest Ethiopia, 2025.

Among six carbapenemase-sparing antibiotics tested against 11 ESBL-producing isolates (five *E. coli* and six *K. pneumoniae*), 90.9% of them were susceptible to cefotetan, while they showed resistance to aztreonam. Meanwhile, 45.5% ESBL producers exhibited resistance to nitrofurantoin (Fig 3).

### Antibiotic susceptibility profiles of *E. coli* and *K. pneumoniae*

Among 21 *E. coli* and 38 *K. pneumoniae* tested, the highest resistance level was observed against ampicillin (71.4%) and amoxicillin-clavulanic acid (71.1%), respectively. Furthermore, 28.6% of *E. coli* and 18.4% of *K. pneumoniae* were resistant to cefotaxime (extended-spectrum cephalosporin). On the other hand, all *E. coli* and 37 *K. pneumoniae* (97.4%) were susceptible to amikacin. Unlike *E. coli* having 100% susceptibility, two *K. pneumoniae* (5.3%) were resistant to carbapenems (Table 3).

**Table 3:** Antimicrobial susceptibility profile of *E. coli* and *K. pneumoniae* isolated from raw bulk cow milk collected from dairy development and marketing cooperative member farms in Northwest Ethiopia, 2025.

Antibiotic category	Antibiotics tested	Resistant *E. coli*: n (%)	Resistant *K. pneumoniae*: n (%)
**Penicillins**	Ampicillin	15 (71.4)	NA
**Penicillin-β-lactamase inhibitor**	Amoxicillin-clavulanic acid	11 (52.4)	27 (71.1)
**Non-extended spectrum cephalosporins (1**^**st**^ **and 2**^**nd**^ **generation)**	Cefazolin	13 (61.9)	21 (55.3)
	Cefuroxime	9 (42.9)	9 (23.7)
**Extended spectrum cephalosporins (3**^**rd**^ **and 4**^**th**^ **generation)**	Ceftazidime	5 (23.8)	5 (13.2)
	Cefotaxime	6 (28.6)	7 (18.4)
**Carbapenems**	Meropenem	0	2 (5.3)
	Imipenem	0	2 (5.3)
**Aminoglycosides**	Gentamicin	1 (4.8)	1 (2.6)
	Amikacin	0	1 (2.6)
**Fluoroquinolones**	Ciprofloxacin	5 (23.8)	6 (15.8)
**Tetracyclines**	Tetracycline	15 (71.4)	17 (44.7)
**Folic acid synthesis inhibitors**	Co-trimoxazole	7 (33.3)	13 (34.2)
**Phenicols**	Chloramphenicol	3 (14.3)	3 (7.9)

**Note:** NA → Not applicable.

Among two carbapenem-resistant *K. pneumoniae* identified in this study, one isolate was carbapenemase producer. Both isolates showed resistance to all β-lactam, fluoroquinolone, tetracycline, and folic acid synthesis inhibitor antibiotics tested. Among the two isolates, carbapenemase-mediated carbapenem-resistant isolates also showed resistance to aminoglycosides. However, both isolates were susceptible for chloramphenicol.

**Fig 4:**
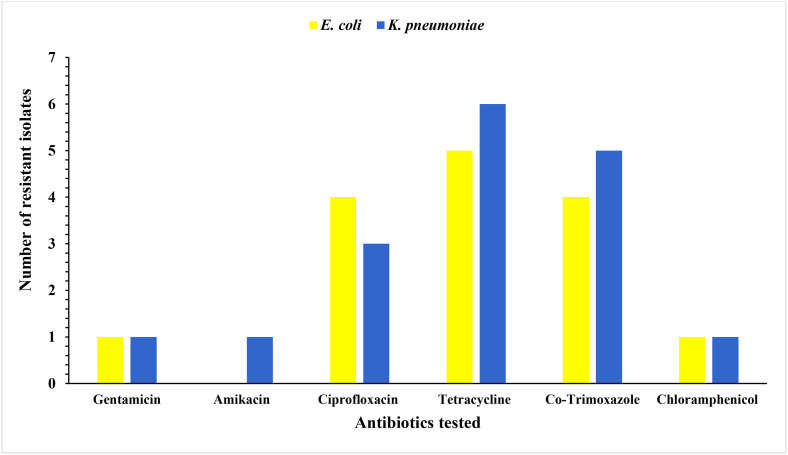
Co-resistance level of ESBL-producing *E. coli* and *K. pneumoniae* isolated from raw bulk cow milk against non-β-lactam classes of antibiotics, Northwest Ethiopia.

All ESBL-producing *E. coli* and *K. pneumoniae* were co-resistant to tetracycline. However, only one isolate from both ESBL-producing *E. coli* and *K. pneumoniae* showed co-resistance to chloramphenicol. ESBL-producing *E. coli* did not show any co-resistance to amikacin (Fig 4).

### Multidrug resistance profile of *E. coli* and *K. pneumoniae*

The overall MDR rate of both *E. coli* and *K. pneumoniae* was 45.8% (27/59). Though not significantly associated (Chi-Square *p* = 0.192), higher MDR was documented for *E. coli* (57.1%) than *K. pneumoniae* (39.5%). Among MDR isolates, half of *K. pneumoniae* (8/15, 53.3%) and *E. coli* (6/12, 50%) were resistant to six or more antibiotics tested (Table 4).

**Table 4:** Multidrug resistance levels of *E. coli* and *K. pneumoniae* isolated from raw bulk cow milk against different groups of antibiotics, Northwest Ethiopia.

Resistance pattern	*E. coli*: n (%)	*K. pneumoniae*: n (%)
AMP*, AMC, TE	1 (8.33)	-^⁑^
AMC, KZ, TE	–	3 (20)
AMC, KZ, COT	–	1 (6.66)
KZ, TE, COT, C	1 (8.33)	–
AMC, KZ, TE, COT	–	2 (13.4)
AMP, AMC, TE, C	1 (8.33)	–
AMC, KZ, CIP, TE, COT	–	1 (6.66)
AMP, AMC, KZ, CXM, TE	2 (16.66)	–
AMP, AMC, KZ, CXM, COT	1 (8.33)	–
AMP, KZ, CXM, CTX, CIP, TE	1 (8.33)	–
AMC, KZ, CXM, CTX, TE, COT	–	1 (6.66)
AMC, KZ, CXM, CIP, TE, COT, C	–	1 (6.66)
AMC,KZ, CXM, CAZ, CTX, TE, C	–	1 (6.66)
KZ, CXM, CAZ, CTX, CIP, TE, COT	–	1 (6.66)
AMC, KZ, CXM, CTX, CIP, TE, COT, C	–	1 (6.66)
AMP*, AMC, KZ, CXM, CAZ, CTX, TE, COT	1 (8.33)	1 (6.66)
AMP, AMC, KZ, CXM, CAZ, CTX, CIP, TE, COT	2 (16.66)	–
AMC, KZ, CXM, CTX, MRP, IMP, CIP, TE, COT	–	1 (6.66)
AMP, AMC, KZ, CXM, CAZ, CTX, CIP, TE, COT, C	1 (8.33)	–
AMP, AMC, KZ, CXM, CAZ, CTX, CN, CIP, TE, COT	1 (8.33)	–
AMC, KZ, CXM, CAZ, CTX, MRP, IMP, CN, AK, CIP, TE, COT	–	1 (6.66)
**Total**	**12 (57.1)**	**15 (39.5)**

**Note**: Ampicillin is tested only for *E. coli*, ⁑→ since *K. pneumoniae* is not tested for AMP, *K. pneumoniae* resistant to two antibiotics (AMC, TE) will not be considered MDR, “-“ → no MDR. AK→ amikacin, AMC→ amoxycillin-clavulanic acid, AMP → ampicillin, CAZ→ ceftazidime, C→ chloramphenicol, CIP→ ciprofloxacin, COT → Co-Trimoxazole, CTX→ cefotaxime, CXM→ cefuroxime, GM→ gentamicin, IPM→ imipenem, KZ→ cefazolin, MRP→ meropenem, TE→ tetracycline.

The MDR profile showed significant variation (Chi-Square *p* = 0.001) among dairy cooperatives, where the highest and the lowest MDR *E. coli* and *K. pneumoniae* were isolated from DC-D (84.2%) and DC-C (12.5%), respectively. Significantly (*p* = 0.003) higher numbers of *E. coli* and *K. pneumoniae* were isolated from milk samples collected from DC members who share houses with their animals were MDR than those who did not share houses. Similarly, significantly higher (84.2%) MDR *E. coli* and *K. pneumoniae* were isolated from milk samples collected from dairy cooperative members who bought drugs from stores without prescription than those who got drugs from house-to-house service given by animal health professionals (27.5%) (*p* < 0.001) (Table 5).

**Table 5:** Description of MDR *E. coli* and *K. pneumoniae* isolated from raw bulk cow milk in different aspects of the dairy farm and farm owners, Northwest Ethiopia, 2025.

Variables	MDR: n (%)		*p*-value
	Yes	No	
Dairy Cooperatives			<0.001*
DC-A (n = 13)	4 (30.8)	9 (69.2)	
DC-B (n = 19)	6 (31.6)	13 (68.4)	
DC-C (n = 8)	1 (12.5)	7 (87.5)	
DC-D (n = 19)	16 (84.2)	3 (15.8)	
Sharing the same house with animals			0.003*
Yes (n = 7)	7 (100)	0	
No (n = 52)	20 (38.5)	32 (61.5)	
Presence of bedding material in cattle barn			0.008^♦^
Present (n = 35)	11 (31.4)	24 (68.6)	
Absent (n = 24)	16 (66.7)	8 (33.3)	
Source of drugs for animals			<0.001^♦^
Animal drug store without prescription (n = 19)	16 (84.2)	3 (15.8)	
House-to-house from professionals (n = 40)	11 (27.5)	29 (72.5)	
Presence of sick animal during data collection			0.508^♦^
Yes (13)	7 (53.8)	6 (46.2)	
No (46)	20 (43.5)	26 (56.5)	
Drinking water source for cattle			0.036^♦^
Tape water only (N = 14)	3 (21.4)	11 (78.6)	
River/spring (N = 45)	24 (53.3)	21 (46.7)	
Free grazing feeding			0.026^♦^
Yes (30)	18 (60)	12 (40)	
No (29)	9 (31)	20 (69)	
Human and animal leftover drug disposal system			0.319*
Toilet (n = 10)	3 (30)	7 (70)	
With animal manure/river (n = 49)	24 (49)	25 (51)	
**Total**	**27 (45.8)**	**32 (54.2)**	

**Note**: ♦ → P-value obtained from Chi-Square test, * → P-value obtained from Fisher’s exact test, DC-A→ Dairy Cooperative-A, DC-B→ Dairy Cooperative-B, DC-C→ Dairy Cooperative, DC-D→ Dairy Cooperative-D

### Factors associated with positive culture results of bulk cow milk for *E. coli* and/or *K. pneumoniae*

Eighteen independent variables were considered for bivariable binary logistic regression, where 14 of them became eligible for multivariable binary logistic regression (*p* ≤ 0.25). Before performing multivariable binary logistic regression, we assessed multicollinearity among independent variables using the variance inflation factor (VIF). All variables had VIF value less than 2, indicating an acceptable level of multicollinearity. Five variables including possessing pet animals (AOR = 6.53, 95% CI: 1.39–30.7) and using calabash as milk pail (AOR = 7.37, 95% CI: 1.45–37.49)) were significantly associated with positive culture results of milk for E. coli and/or K. pneumoniae. Though the presence of sick animals in the herd and teat injury in lactating cows were significantly associated in bi-variable logistic regression, they failed to show a significant association in multivariable analysis. Raw bulk cow milk samples collected from members of DC-D and DC-B were 10.1 and 2.9 times more likely to be contaminated with potential pathogenic *E. coli* and/or *K. pneumoniae*, respectively than samples collected from DC-A. Similarly, raw bulk cow milk samples collected from dairy cooperative members having pet animals were 6.5 times more likely to be contaminated with potential pathogenic with *E. coli* and/or *K. pneumoniae* than samples collected from dairy cooperative members who did not have pet animals (Table 6).

**Table 6:** Bivariable and multivariable binary logistic regression analysis of factors associated with positive culture results of raw bulk cow milk samples collected from dairy development and marketing cooperative members in Northwest Ethiopia, 2025.

Variable	Culture result: n (%)		*p*-value	COR (95% CI)	*p*-value	AOR (95% CI)
	Positive	Negative				
Dairy Cooperatives						
DC-A (104)	12 (11.5)	92 (88.5)	1			
DC-B (72)	18 (25)	54 (75)	0.022	2.56 (1.14–5.71)	0.030*	2.894 (1.11–7.54)
DC-C (55)	8 (14.5)	47 (85.5)	0.587	1.31 (0.50–3.41)	0.757	1.20 (0.38–3.82)
DC-D (26)	16 (61.5)	10 (38.5)	<0.001	12.27 (4.54–33.11)	0.001*	10.14 (2.47–41.60)
Educational status						
No formal education (105)	31 (29.5)	74 (70.5)	0.006	2.35 (1.28–4.33)	0.088	2.06 (0.90–4.74)
Attended formal education (152)	23 (15.1)	129 (84.9)	1			
Occupational status						
Has additional work (46)	16 (34.8)	30 (65.2)	0.013	2.43 (1.21–4.90)	0.007*	4.17 (1.49–11.67)
Farming only(211)	38 (18)	173 (82)	1			
Have cowherd						
No (147)	39 (26.5)	108 (73.5)	0.013	2.29 (1.19–4.41)	0.174	1.74 (0.78–3.84)
Yes (110)	15 (13.6)	95 (86.4)	1			
Herd size						
Large (58)	18 (31)	40 (69)	0.035	2.04 (1.05–3.95)	0.014*	3.21 (1.26–8.18)
Small (199)	36 (18.1)	163 (81.9)	1			
Have a separate cattle barn						
No (13)	6 (46.2)	7 (53.8)	0.031	3.50 (1.13–10.89)	0.087	4.18 (0.81–21.47)
Yes (244)	48 (19.7)	196 (80.3)	1			
Sick animal in the herd						
There is a sick animal (25)	10 (40)	15 (60)	0.018	2.85 (1.20–6.76)	0.609	1.42 (0.37–5.47)
No sick animal (232)	44 (19)	188 (81)	1			
Have pet animals						
Yes (200)	52 (26)	148 (74)	0.002	9.66 (2.78–41.02)	0.017*	6.53 (1.39–30.7)
No (57)	2 (3.5)	55 (96.5)	1			
Number of lactating cows						
More than one cow (n = 207)	47 (22.7)	160 (77.3)	0.180	1.80 (0.762–4.28)	0.633	1.30 (0.45–3.74)
One cow (n = 50)	7 (14)	43 (86)	1			
A lactating cow has teat injury						
Yes (n = 43)	14 (32.6)	29 (67.4)	0.045	2.10 (1.02–4.33)	0.500	1.44 (0.50–4.13)
No (n = 214)	40 (18.7)	174 (81.3)	1			
Number of milker/farm						
More than one (n = 205)	49 (23.9)	156 (76.1)	0.03	2.95 (1.11–7.84)	0.275	2 (0.58–5.15)
One (n = 52)	5 (9.6)	47 (90.4)	1			
Milk pail type						
Calabash (n = 17)	6 (35.3)	11 (64.7)	<0.001	8.40 (2.95–23.95)	0.016*	7.37 (1.45–37.49)
Plastic/metal (n = 240)	43 (17.9)	197 (82.1)	1			
Animal free-grazing						
Yes (n = 76)	27 (35.5)	49 (64.5)	<0.001	3.14 (1.67–5.86)	0.766	1.14 (0.48–2.72)
No (n = 181)	27 (14.9)	154 (85.1)	1			
Wash a cow body regularly						
After a month (n = 204)	48 (23.5)	156 (76.5)	0.058	2.41 (0.97–5.98)	0.326	1.73 (0.58–5.15)
At least per month (n = 53)	6 (11.3)	47 (81.1)	1			
Total	54 (21)	203 (79)				

Note: AOR→ Adjusted Odds Ratio, CI→ Confidence Interval, COR→ Crud Odds Ratio, * → significantly associated

## Discussion

In this study, we found that 21% of milk samples were contaminated with *E. coli* and/or *K. pneumoniae*, with a notable prevalence of ESBL-producing *E. coli* (23.8%) and *K. pneumoniae* (15.8%), along with 2.6% carbapenemase-producing *K. pneumoniae*. The study also found that nearly half of the isolates were multidrug-resistant (MDR), with significant variation across dairy cooperatives. Factors including large herd size, calabash-made milk pail use, and presence of pet animals were significantly associated with milk culture positive results.

Milk collected from healthy cows can have diverse groups of microorganisms including *E. coli* and *K. pneumoniae* with different levels of abundance emanating from calves through retrograde transfer, external environment and endogenous sources via entero-mammary and rumen-mammary pathways [49–51]. Microbial contamination level of milk might increase further starting from milking to the point where it reaches consumers [49]. The finding of 21% milk contamination level by these pathogenic bacteria, well-known to cause diseases in humans and animals [52–54], might emanate from milk microbiota [49], diseased animals [55,56], and various sources like contamination with animal dung, bedding material, water source, human fecal matter, milking equipment, and unhygienic milk transportation to the dairy cooperatives [57,58]. Given that the consumption of raw bulk cow milk is a common practice [30], our findings suggest that it may act as a source of pathogenic bacteria responsible for milk-borne disease [59].

We observed variation in prevalence among dairy cooperatives for *E. coli* and *K. pneumoniae*. There was a significantly higher prevalence of *E. coli* and *K. pneumoniae* in DC-D compared to other dairy cooperatives. This observation is consistent with previous reports from Ethiopia [60] and China [61], which highlighted notable variations in bacterial contamination among dairy farms. Sociodemographic data from this study revealed that all DC-D members reside in rural areas, with more than half lacking formal education. These characteristics may limit access to and understanding of food safety training, a critical component for maintaining hygienic milk handling practice [62]. Moreover, a significantly higher number of DC-D members reported using a traditional calabash-made milk pail, an equipment which was found in this study to be significantly associated with bacterial contamination. The porous nature and cleaning challenges associated with calabash containers may further elevate the risk of microbial proliferation, thereby contributing to the increased prevalence of *E. coli* and *K. pneumoniae* in DC-D milk samples. Other factors like dirty milk pail, not washing hands before milking, unhygienic animal barn, water source, and poor animal hygiene might have a role in the observed variations [37,63].

Notably, the number of *K. pneumoniae* isolates in this study was nearly double that of *E. coli*, a trend commonly reported in clinical isolates [64]. This finding contrasts with earlier studies on milk samples, which generally reported a higher prevalence of *E. coli* [25,40,65–68]. A shift may reflect a growing role of *K. pneumoniae* in both clinical and subclinical mastitis, particularly in developing countries and high-density regions [56]. Moreover, the clonal dissemination of *K. pneumoniae* from pet animals may further explain its increased detection in dairy farms [69]. These findings underscore the need for improved hygiene education, access to sanitary milking equipment, and monitoring of emerging pathogens to enhance milk safety in rural dairy sectors. The lowest collection of milk per cow in DC-D with the highest *K. pneumoniae* prevalence (42.3%) might support the frequent occurrence of this same bacterium. The high prevalence of *K. pneumoniae* may also be caused by the bacterium’s preferred survival rate in warm, humid conditions [9,70] making our study area suitable environment [36] as well as variables influencing the relative abundance of bacteria in raw milk such as geographical location, free grazing, sampling method [49,71], and bedding material [58].

In this study, the prevalence of *E. coli* was 8.2%. A significantly higher prevalence of *E. coli* ranging from 34.4% to 57.1% was reported in other parts of Ethiopia [27,40,72], China [73,74], and in Malaysia [75]. This could be because of variations in geographic location, farm sanitation, health and housing conditions of the cattle, and pre-enrichment of milk samples in certain studies before culturing.

Further emphasizing antimicrobial resistance as a public health concern, this study found that 18.6% of raw bulk cow milk samples were contaminated with ESBL-producing *E. coli* and/or *K. pneumoniae*, with individual prevalence rates of 23.8% for *E. coli* and 15.8% for *K. pneumoniae*. These results support earlier findings that raw milk can act as a reservoir for zoonotic, antibiotic-resistant pathogens [25]. While data on ESBL prevalence in raw milk are scarce, the rate of ESBL-producing *E. coli* observed in our study was lower than that reported in a related Ethiopian study [40] but higher than Malaysia [75]. The detection of ESBL-producing bacteria, especially *K. pneumoniae*, which, despite its lower prevalence, is associated with high mortality [64], raises serious food safety and public health concerns. These findings highlight the urgent need for targeted interventions, including hygiene training, promotion of sanitary milking equipment, and surveillance of antimicrobial resistance in dairy supply chains, particularly in rural settings.

All ESBL-producing *E. coli* and *K. pneumoniae* in this study were resistant to at least six of the tested antibiotics. This could be explained by the frequent co-occurrence of ESBL coding genes and other antibiotic resistance genes in a single mobile genetic element [76]. The high resistance level relates to commonly used antibiotics in dairy farms [33,44,77] and poses an added challenge to properly manage the disease caused by the same bacteria, e.g., severe mastitis [66]. These might be due to easy accessibility of antibiotics without prescription [44,77] facilitating their resistance [65].

Antimicrobial susceptibility testing was done for all ESBL-producing *E. coli* and *K. pneumoniae* against carbapenem-sparing antibiotics including piperacillin-tazobactam, nitrofurantoin, cefepime and cefotetan [78–80]. Sixty per cent and 40% of ESBL-producing *E. coli* and *K. pneumoniae* in this study were resistant to cefepime (fourth-generation cephalosporin), respectively. This was significantly higher than a study in Malaysia [75] and Bangladesh [81] reporting 0% and 2% resistance rates, respectively for *E. coli*. This might be partly attributed to an increasing trend in community-based consumption of 4^th^ generation cephalosporin in Ethiopia [82]. Although all ESBL-producing *E. coli* were susceptible to cefotetan, 16.7% of *K. pneumoniae* were resistant, which is higher than the 1.8% resistance level reported for the same bacterium in clinical samples [83]. ESBL-producing *E. coli* and *K. pneumoniae* in this study showed a higher resistance rate to cefoxitin than cefotetan. This trend was also observed in *K. pneumoniae* isolated from clinical samples [83]. The resistance level of ESBL-producing *E. coli* to cefoxitin in this study was 20%, fairly comparable with a report from Bangladesh (15%) [81]. Overall, the resistance rate of ESBL-producing *E. coli* and *K. pneumoniae* for carbapenem-spearing treatment options, especially to cefepime and cefotetan, is a great concern.

The prevalence of carbapenemase producing *K. pneumoniae* in this study was 2.6% with no carbapenemase producing *E. coli.* This was higher than a study from Italy [84] reporting 0.4%. The carbapenemase producing *K. pneumoniae* in this study was resistant to 20 antibiotics tested including ceftazidime-avibactam and meropenem-vaborbactam, which the WHO classified as “reserved” antibiotics for human use in 2022 [85]. This can be a significant health threat given the detection of high-risk clones of carbapenemase-producing *K. pneumoniae* in milk [25] coupled with limited treatment options, sever disease outcome and possible inter-species transmission between human and animal [56,86–88]. The possible cause of carbapenem resistance *K. pneumoniae* in milk may be attributed to the inappropriate use of carbapenems intended for human medicine in dairy farms despite their ban [89] or the transfer of the resistant bacteria from carrier workers, animals or the surrounding environment.

Raw milk is a significant source of antimicrobial resistant bacteria and their coding genes [90]. Understanding the extent of the problem is key to preventing human transmission in countries like Ethiopia where raw milk consumption is practiced [30,31]. Ampicillin, tetracycline and co-trimoxazole are among the commonly used antibiotics in Ethiopian dairy farms [44,77]. In this study, *E. coli* showed the highest level of resistance to both ampicillin and tetracycline (71.4%). This result also supports previous studies in Ethiopia [40,60] reporting higher levels of resistance to these same antibiotics. Meanwhile, the *E. coli* resistance rate to ampicillin in this study was higher than reports from Zambia, 50% [24], China, 46.3% [73], and Romania, 26.2% [42]. Likewise, the present study documented higher levels of tetracycline resistance by *E. coli* when compared to studies in Africa [24], Asia [27,73], and Europe [42]. Close to half *K. pneumoniae* isolates (44.7%) were resistant to tetracycline which was comparable with the resistance rate of the same bacteria isolated from a cow with mastitis [91]. One-third of *E. coli* and *K. pneumoniae* isolates in this study were resistant to co-trimoxazole. Though a higher resistance rate of *E. coli* to co-trimoxazole was reported in other parts of Ethiopia [40,60] the result of this study was significantly higher when compared to studies in Zambia [24], China [73,92] and Romania [42]. High resistance level of *E. coli* and *K. pneumoniae* to ampicillin, tetracycline, and co-trimoxazole in this study might be due to low awareness level about antibiotic use and AMR among livestock producers [33], widespread use of antibiotics for growth promotion, lack of diagnostic tools and sub-standard antibiotics in Amhara regional state [44]. Having a history of buying drugs without prescription by all dairy cooperative members in this study might also contribute to the observed antibiotic resistance.

In this study, 89.8% of *E. coli* and *K. pneumoniae* including carbapenem resistant isolates were susceptible to chloramphenicol. This might be due to decreased use of the antibiotics associated with their side effect and limited availability [93]. A declining trend in chloramphenicol resistance was also reported from clinical bacterial isolates including Ethiopia [94,95].

Overall, 45.8% of the isolates were MDR with 51.9% resistance to at least 6 antibiotics. This finding augments a previous study reporting the widespread presence of MDR bacteria isolated from human, animal, food and environmental samples in Ethiopia [96]. The finding in this study was higher than the MDR rate of *E. coli* and *K. pneumoniae* isolated from clinical samples in Amhara Regional State including our study area [94]. The transmission of these MDR bacteria from farm workers and/or the surrounding environment to raw milk may have been facilitated by inadequate sanitary practices in Ethiopian dairy farms [97]. Factors such as sharing houses with animals, using river water for animal drinking, allowing cattle for free grazing and the absence of bedding material were significantly associated with MDR bacteria isolation from milk samples in this study. These support the existence of favorable ground for the spread of MDR bacteria among farm workers, the environment and cow milk. Furthermore, the majority of the genes in *K. pneumoniae* and *E. coli* that code for resistance to different antibiotic classes are found in mobile genetic elements which facilitate their transfer between clinical and non-clinical bacterial isolates calling for a comprehensive approach in the fight against AMR [26,98].

Contamination of raw cow milk with potentially pathogenic *E. coli* and *K. pneumoniae* in this study were significantly associated with large herd size, presence of pet animals in the farm, involvement of farm owners in other work alongside dairy farming, and using a milk pail made of calabash. Other studies in Ethiopia [60] and China [99] revealed similar substantial relationships on herd size and geographical area, respectively. However, a distinct herd size finding was reported in central Ethiopia [60] which might be attributed to differences in sample size and classification of herds as small and large. Compared to calabash containers, milk samples collected from containers made of stainless steel or plastic have significantly lower levels of *E. coli* and *K. pneumoniae* contamination. This could be because calabash containers are porous and more difficult to clean than other types of containers, which makes them an ideal environment for bacterial growth. Though not statistically significant, a similarly lower level of milk contamination was reported in Ethiopia [60] from samples collected in stainless steel containers than from the other types.

As a limitation of our study, strain level identification of *E. coli* and *K. pneumoniae* were not done. Moreover our study did not also identified the type, and variants of ESBL and carbapenemase enzymes. However there is variation in virulence, and transmission of *E. coli* and *K. pneumoniae* depending on bacterial strains, as well as types and variants of enzymes [100,101].

## Conclusions

The prevalence of *E. coli* and/or *K. pneumoniae* in raw bulk cow milk from dairy cooperatives in Northwest Amhara was low compared to previous reports from Ethiopia. Meanwhile, the prevalence varied among cooperatives, with milk collected from DC-D resulted in the highest prevalence. The number of *K. pneumoniae* isolates was nearly double that of *E. coli*. High levels of ESBL-producing *E. coli* and *K. pneumoniae* prevalence, with alarming MDR levels, were found. The raw cow milk may therefore represent a significant source of zoonotic MDR *E. coli* and *K. pneumoniae*, including carbapenemase and ESBL producing isolates resistant to carbapenem-sparing antibiotics, primarily cefepime and cefotetan. Large herd size, having pet animals, using a calabash-made milk pail, and engaging in other revenue-generating activities in addition to dairy farming were significantly associated with raw bulk cow milk culture positive results. Known sanitary measures including keeping the hygiene of milking process, and the herd environment should be in place by all dairy cooperative members with immediate attention for DC-D. Dairy cooperative members should also manage their herds appropriately, devote adequate time to farming, and ensure proper care of their pet animals. Integrated AMR surveillance, antimicrobial stewardship in veterinary medicine and training of dairy cooperative members on hygienic practices in the milk value chain are helpful interventions. Furthermore, high-resolution molecular studies are needed to elucidate reservoirs, dominant sequence types, and variants of ESBL-producing *E. coli* and *K. pneumoniae* in milk.
